# Acipimox in Mitochondrial Myopathy (AIMM): study protocol for a randomised, double-blinded, placebo-controlled, adaptive design trial of the efficacy of acipimox in adult patients with mitochondrial myopathy

**DOI:** 10.1186/s13063-022-06544-x

**Published:** 2022-09-20

**Authors:** The AIMM Trial Group:, Alaa Abouhajar, Lisa Alcock, Theophile Bigirumurame, Penny Bradley, Laura Brown, Ian Campbell, Sylvia Del Din, Julie Faitg, Gavin Falkous, Gráinne S. Gorman, Rachel Lakey, Robert McFarland, Jane Newman, Lynn Rochester, Vicky Ryan, Hesther Smith, Alison Steel, Renae J. Stefanetti, Huizhong Su, Robert W. Taylor, Naomi J.P. Thomas, Helen Tuppen, Amy E. Vincent, Charlotte Warren, Gillian Watson

**Affiliations:** 1 Newcastle Clinical Trials Unit, 1-4 Claremont Terrace, Newcastle University, Newcastle upon Tyne, NE2 4AE UK; 2 Brain and Movement Research Group, Clinical Ageing Research Unit, Translational and Clinical Research Institute, Newcastle University, Newcastle upon Tyne, NE4 5PL UK; 3 Population Health Sciences Institute, Newcastle University, Ridley 1 Building, Newcastle upon Tyne, NE1 7RU UK; 4 Pharmacy Directorate, Newcastle Upon Tyne Hospitals NHS Foundation Trust, Freeman Hospital, Freeman Road, Newcastle Upon Tyne, NE7 7DN UK; 5 Wellcome Centre for Mitochondrial Research, Translational and Clinical Research Institute, Faculty of Medical Sciences, Newcastle University, Newcastle Upon Tyne, NE2 4HH UK; 6 NHS Highly Specialised Service for Rare Mitochondrial Disorders, Newcastle Upon Tyne Hospitals NHS Foundation Trust, Newcastle Upon Tyne, NE1 4LP UK

**Keywords:** Acipimox, Adenosine triphosphate, Mitochondrial disease, Mitochondria, Myopathy, Randomised controlled trial

## Abstract

**Background:**

Mitochondrial disease is a heterogenous group of rare, complex neurometabolic disorders. Despite their individual rarity, collectively mitochondrial diseases represent the most common cause of inherited metabolic disorders in the UK; they affect 1 in every 4300 individuals, up to 15,000 adults (and a similar number of children) in the UK. Mitochondrial disease manifests multisystem and isolated organ involvement, commonly affecting those tissues with high energy demands, such as skeletal muscle. Myopathy manifesting as fatigue, muscle weakness and exercise intolerance is common and debilitating in patients with mitochondrial disease. Currently, there are no effective licensed treatments and consequently, there is an urgent clinical need to find an effective drug therapy.

**Aim:**

To investigate the efficacy of 12-week treatment with acipimox on the adenosine triphosphate (ATP) content of skeletal muscle in patients with mitochondrial disease and myopathy.

**Methods:**

AIMM is a single-centre, double blind, placebo-controlled, adaptive designed trial, evaluating the efficacy of 12 weeks’ administration of acipimox on skeletal muscle ATP content in patients with mitochondrial myopathy. Eligible patients will receive the trial investigational medicinal product (IMP), either acipimox or matched placebo. Participants will also be prescribed low dose aspirin as a non-investigational medical product (nIMP) in order to protect the blinding of the treatment assignment.

Eighty to 120 participants will be recruited as required, with an interim analysis for sample size re-estimation and futility assessment being undertaken once the primary outcome for 50 participants has been obtained. Randomisation will be on a 1:1 basis, stratified by Fatigue Impact Scale (FIS) (dichotomised as < 40, ≥ 40). Participants will take part in the trial for up to 20 weeks, from screening visits through to follow-up at 16 weeks post randomisation.

The primary outcome of change in ATP content in skeletal muscle and secondary outcomes relating to quality of life, perceived fatigue, disease burden, limb function, balance and walking, skeletal muscle analysis and symptom-limited cardiopulmonary fitness (optional) will be assessed between baseline and 12 weeks.

**Discussion:**

The AIMM trial will investigate the effect of acipimox on modulating muscle ATP content and whether it can be repurposed as a new treatment for mitochondrial disease with myopathy.

**Trial registration:**

EudraCT2018-002721-29. Registered on 24 December 2018,

ISRCTN 12895613. Registered on 03 January 2019, https://www.isrctn.com/search?q=aimm

**Supplementary Information:**

The online version contains supplementary material available at 10.1186/s13063-022-06544-x.

## Administrative information

Note: the numbers in curly brackets in this protocol refer to SPIRIT checklist item numbers. The order of the items has been modified to group similar items (see http://www.equator-network.org/reporting-guidelines/spirit-2013-statement-defining-standard-protocol-items-for-clinical-trials/).

**Table:** 

Title {1}	**Acipimox in Mitochondrial Myopathy (AIMM): study protocol for a randomised, double-blinded, placebo-controlled, adaptive design trial of the efficacy of acipimox in adult patients with mitochondrial myopathy**
Trial registration {2a and 2b}.	EudraCT: 2018-002721-29 ISRCTN: 12895613
Protocol version {3}	The AIMM trial is currently working to protocol version 5.0, dated 3^rd^ June 2021
Funding {4}	This trial is funded by the Medical Research Council Biomedical Catalyst Developmental Pathway Funding Stream (DPFS) (funder reference MR/R006458/1). The funder did not contribute to this manuscript, but is acknowledged within the publication.
Author details {5a}	**The AIMM Trial Group** In alphabetical order: Alaa Abouhajar^1^ Lisa Alcock^2^ Theophile Bigirumurame^3^ Penny Bradley^4^ Laura Brown^5^ Ian Campbell^4^ Silvia Del Din^2^ Julie Faitg^5^ Gavin Falkous^5^ Gráinne S. Gorman^5,6^ Rachel Lakey^1^ Robert McFarland^5,6^ Jane Newman^5^ Lynn Rochester^2^ Vicky Ryan^3^ Hesther Smith^4^ Alison Steel^1^ Renae J. Stefanetti^5^ Huizhong Su^5^ Robert W. Taylor^5,6^ Naomi J.P. Thomas^5^ (corresponding author) Helen Tuppen^5^ Amy E. Vincent^5^ Charlotte Warren^5^ Gillian Watson^1^ 1. Newcastle Clinical Trials Unit, 1-4 Claremont Terrace, Newcastle University Newcastle upon Tyne NE2 4AE 2. Brain and Movement Research Group, Clinical Ageing Research Unit, Translational and Clinical Research Institute, Newcastle University, Newcastle upon Tyne, NE4 5PL 3. Population Health Sciences Institute, Newcastle University, Ridley 1 Building, Newcastle upon Tyne, NE1 7RU 4. Pharmacy Directorate, Newcastle Upon Tyne Hospitals NHS Foundation Trust, Freeman Hospital, Freeman Road, Newcastle Upon Tyne, NE7 7DN 5. Wellcome Centre for Mitochondrial Research, Translational and Clinical Research Institute, Faculty of Medical Sciences, Newcastle University, NE2 4HH 6. NHS Highly Specialised Service for Rare Mitochondrial Disorders, Newcastle Upon Tyne Hospitals NHS Foundation Trust, Newcastle Upon Tyne, NE1 4LP
Name and contact information for the trial sponsor {5b}	The Newcastle upon Tyne Hospitals NHS Foundation Trust are the trial Sponsor. Newcastle Clinical Trials Unit, 1-4 Claremont Terrace, Newcastle University Newcastle upon Tyne NE2 4AE
Role of sponsor {5c}	All trial management responsibilities have been delegated by Sponsor to Newcastle Clinical Trials Unit (NCTU), including trial design; review and approval of all patient-facing documentation prior to implementation. Sponsor is responsible for signing off amendments and trial procedures must not be changed without the agreement of the CI, Sponsor and Trial Management Group (TMG) Substantial amendments will be submitted to the appropriate regulatory authorities and will not be implemented until this approval is in place. Sponsor did not contribute to this manuscript but provided review and approval prior to submission for publication.

## Introduction

### Background and rationale {6a}

Mitochondria are ubiquitous, semi-autonomous intracellular organelles that play an essential role in many cellular processes. This includes oxidative phosphorylation (OXPHOS), which harnesses energy generated by a proton electrochemical gradient to synthesise adenosine triphosphate (ATP) from adenosine diphosphate (ADP) and inorganic phosphate, the main source of utilisable energy for intracellular metabolic pathways [1].

Mitochondrial disease is a diverse group of complex neurometabolic disorders characterised by defects in OXPHOS and often affecting organs with high energy demands, including skeletal muscle, heart and brain. Despite their individual rarity, collectively mitochondrial disease represents the most common cause of inherited metabolic disorders in the UK; affecting 1 in every 4300 individuals, up to 15,000 adults (and a similar number of children) in the UK [2].

The complex pathophysiology of mitochondrial disease is exacerbated by the dual genetic control of mitochondrial function. Disease can be caused by pathogenic variants in the nuclear or mitochondrial genomes (mtDNA), and accordingly inheritance patterns can be autosomal, sex-linked or maternal. In patients with pathogenic mtDNA variants, the inheritance and clinical presentation are further complicated by the presence of multiple mtDNA genomes within individual cells, often leading to a mixture of mutated and wild-type genomes, referred to as ‘heteroplasmy’. Variation in mtDNA heteroplasmy influences the extent of cellular dysfunction, but the relationship is unpredictable, and only partially explains variation in expression and phenotype [3].

Myopathy manifesting as fatigue, exercise intolerance, myalgia and muscle weakness is extremely prevalent, and often debilitating, in mitochondrial disease. Affected patients are often unable to work or require costly medical care.

Whilst there have been recent rapid developments in the field of mitochondrial medicine and pivotal steps made in our understanding of the genetic basis of mitochondrial diseases, therapies remain limited and often confined to specific mitochondrial diseases or alleviation of symptoms only [4, 5].

Nutritional supplements and vitamin ‘cocktails’ have been widely used to treat mitochondrial diseases, but the available sparse evidence suggests minimal beneficial effect [6]. Several small trials of exercise training in patients with mitochondrial disease have demonstrated that exercise is a powerful stimulus of mitochondrial biogenesis with consequent improvements in exercise capacity and quality of life [7]. However, the practical application of exercise as a mainstay of treatment may be limited by patient and disease specific factors such as cardiomyopathy or fatigue.

A Cochrane review published in 2012 did not identify any disease-modifying treatments proven to benefit patients with mitochondrial disease [8]. This remains unchanged, although new experimental treatment approaches, including targeting of the autophagic elimination of defective mitochondria, hypoxia, molecular bypass, gene therapy and exploitation of mitochondrial proliferation, are emerging [4, 9]. Whilst these approaches show therapeutic promise, they are in early pre-clinical phases of development and are a considerable way from human trials and routine clinical use.

ClinicalTrials.gov lists 275 trials under mitochondrial disease (no search limitation), of which 175 are interventional and 116 pharmacological (as of November 2021). These studies variously exhibit prevailing difficulties in rare diseases trial design, such as low participant numbers, genotypic and phenotypic heterogeneity and the consequent potential ramifications upon validity and reliability.

Given this paucity of effective treatments, there is an urgent unmet clinical need for new pharmacological strategies. One such strategy is to pharmacologically enhance mitochondrial biogenesis and thereby improve OXPHOS dysfunction. This may be achieved by activating key regulators of mitochondrial biogenesis pathways, including sirtuins (decatalase enzymes, including SIRT-1), PPARƴ co-activator 1 alpha (PGC1α), peroxisome proliferator-activated receptor (PPAR) and AMP-activated protein kinase (AMPK) [10]. ,Our therapeutic target, nicotinamide adenine dinucleotide (NAD^+^), is essential to this activation process; it is a fuel for the sirtuins, and crucial for redox reactions and molecular signalling [11].

Acipimox (5-carboxyl-2-methyl pyrazine 1-oxide) is a niacin derivative and nicotinic acid analogue licensed for the treatment of hyperlipidaemia in non-insulin requiring diabetes mellitus [11]. It has incidentally been demonstrated to potently promote NAD^+^ levels, activate SIRT 1 and enhance mitochondrial gene expression profiles [4, 11, 12]. Acipimox has been consistently reported to have an excellent safety profile.

### Pilot data

Van de Weijer et al. (with Auwerx, Co-Investigator) have demonstrated that acipimox has a direct effect on human skeletal muscle mitochondrial function in patients with type 2 diabetes [12]. Proof of concept data, derived from this work, has facilitated the estimation of participant numbers needed to achieve the trial’s primary objective (change in skeletal muscle ATP content following 12 weeks’ dosing with acipimox compared to matched placebo). The potential benefits of acipimox in patients with impaired mitochondrial function are expected to be relatable to the observed increase in ATP content and increased mitochondrial biogenesis observed in the pilot trial and animal models.

## Objectives {7}

### Aims

The aim of this trial is to determine whether acipimox can be repurposed as a potent stimulator of mitochondrial biogenesis.

Additionally, we aim to further interrogate the complexity of skeletal muscle mitochondrial biogenesis via mass spectrometry, and in a subset of samples, apply proteomics to establish novel biomarker signatures of mitochondrial disease.

We will also employ functional assessments and assess their applicability not only to mitochondrial disease but potentially other neuromuscular diseases in which exercise intolerance and fatigue are prominent.

### Objectives

#### Primary objective

To investigate whether 12 weeks’ treatment with acipimox increases skeletal muscle ATP content in patients with mitochondrial disease and muscle myopathy, compared to a matched placebo.

#### Secondary objectives

To determine change (between baseline and end of 12 weeks’ treatment) in:

1. Health-related quality of life (QoL)
2. Reported perceived fatigue
3. Symptom-limited cardiopulmonary fitness
4. Disease burden
5. Upper and lower limb function, balance and walking
6. Skeletal muscle analyses (ATP/ADP ratio, mitochondrial biogenesis, NAD^+^/NADH ratio, mtDNA copy number, respiratory chain enzyme status, mtDNA heteroplasmy (where appropriate))

## Trial design {8}

AIMM is a single-centre, double blind, placebo-controlled, adaptive trial evaluating the efficacy of 12 weeks of acipimox on the ATP content of skeletal muscle in patients with mitochondrial myopathy.

An interim analysis for the purpose of sample size re-estimation and futility assessment will take place once the primary outcome at week 12 is available for 50 randomised participants.

Following consent, confirmation of eligibility and completion of baseline assessments, participants will be randomised to either acipimox or matched placebo, at a 1:1 ratio. The IMP (250mg capsule) will be taken three times daily and the nIMP, (aspirin 75mg) once daily to protect blinding of the treatment allocation, throughout the 12-week treatment period. Participants will be monitored for adherence to the trial protocol and adverse events via telephone calls at weeks 1, 2, 4, 8, 10 (if applicable) and 16.

## Methods: participants, interventions and outcomes

### Study setting {9}

The trial is open to participants from across the UK, including Northern Ireland. Participants will be identified by the clinical team within the NHS Highly Specialised Service for Rare Mitochondrial Disorders, by Participant Identification Centres (PICs) who will refer potential participants or via self-referral to the site research team. The trial will be run within The Newcastle upon Tyne Hospitals NHS Foundation Trust (NuTH).

### Eligibility criteria {10}

Eligibility will be assessed by a medically qualified doctor. Eligibility criteria are listed in full in Table 1. In brief, inclusion criteria encompass adults (≥16 years) with genetically proven and clinically evident mitochondrial myopathy. Exclusion criteria relate to medical, surgical or psychosocial history, or concomitant medication use that would compromise participant safety or scientific integrity.

**Table 1:** Eligibility criteria

**Inclusion criteria**	
Patients are eligible for the trial if all of the following apply: 1.Must be able to provide full informed consent. 2.Male or female patients ≥ 16 years of age. 3.Patients must fulfil the following: i)Genetically proven diagnosis of mitochondrial disease ii)Evidence of mitochondrial myopathy as confirmed by the investigator 4.Able and willing, in the opinion of the investigator, to comply with all trial requirements. 5.Willing for their GP and Specialist (if applicable), to be informed of their participation in the trial. 6.Be on a stable dose of any current regular medication for at least four weeks prior to trial entry.	
**Exclusion criteria**	
Patients are excluded from the trial if any of the following apply: 1.Patients who are currently participating or have participated in a clinical trial of an investigational medicinal product within the 12-week period prior to the date of informed consent. 2.Patients who have had an elective or emergency admission to hospital within the 4-week period prior to the date of informed consent. 3.Patients with other known uncontrolled medical problems, which, in the opinion of the investigator, would preclude participation in the trial. 4.Patients who are: a. Pregnant b. Breast feeding c. Of childbearing potential with a positive urine pregnancy test prior to starting trial IMP d. Male or female of childbearing potential unwilling to abstain or to use contraception throughout the trial (postmenopausal women must be amenorrhoeic for at least 12 months to be considered of non-childbearing potential). 5.Patients with moderate to severe renal impairment (creatinine clearance via eGFR < 60 ml/min/1.73m^2^). 6.Patients with a screening AST, ALT or Gamma GT result of more than 3 times the upper limit of normal. 7.Patients with a platelet count of < 50 platelets/l of blood 8.Patients on treatment with methotrexate or other immunosuppressant medications. 9.Patients with active known peptic ulcer or history of recurrent ulceration. 10.Patients on treatment with warfarin, clopidogrel, regular high-dose (≥300 mg OD) aspirin or other anticoagulant medications, which in the opinion of the investigator precludes entry into the trial. Patients receiving high-dose aspirin who are able to come off aspirin for a period of 72 h prior to any muscle biopsy sample will be eligible to participate. 11.Patients with a medical history, which in the opinion of the investigator contraindicates the use of low-dose aspirin. 12.Patients who are already taking acipimox. 13.Patients who are taking niacin derivatives. 14.Patients with an elective hospital admission scheduled during the trial period, which in the opinion of the investigator would preclude participation. 15.Patients who may be allergic/unable to take any of the constituent ingredients of the IMP or placebo	

### Who will take informed consent? {26a}

A medically qualified person will take consent prior to any study activity. In cases where a participant is unable to sign or initial the consent form due to weakness or ataxia resulting from their condition, consent will be confirmed orally in the presence of an independent witness who will sign and date the consent form on behalf of the participant.

### Additional consent provisions for collection and use of participant data and biological specimens {26b}

During the consent process, participants will be offered the option of any remaining tissue samples being stored in the Newcastle Mitochondrial Research Biobank (REC Ref: 16/NE/0267), an ethically approved research tissue bank within The Newcastle upon Tyne Hospitals NHS Foundation Trust and Newcastle University. Participants can further specify consent for stored samples being used in animal research or commercial research

### Interventions

#### Explanation for the choice of comparators {6b}

Participants will be randomised to receive acipimox 250 mg oral capsules or matched placebo capsules (IMP) three times daily. This is above the threshold at which the effects on mitochondrial function have been observed; doses of up to 1200mg have been well tolerated according to the product specification [12]

#### Intervention description {11a}

Participants will take their IMP with, or just after a meal, with the first dose of the day taken 20–30 min after the nIMP. No dose modifications are permitted during the treatment period.

The product will be packed and labelled in a blinded fashion into blister strips. The matched placebo will be manufactured to ensure that it is consistent with acipimox in appearance, taste and smell, labelling and packaging. Labelling will comply with Annex 13 of Good Manufacturing Practice.

#### Criteria for discontinuing or modifying allocated interventions {11b}

Participants will have the right to discontinue their allocated trial medication at any time. The participant is not required to provide a reason; however, they will be encouraged to do so to ensure that information relevant to the trial design, medication tolerability and efficacy is collected where possible.

The investigator/treating clinician may discontinue a participant’s allocated trial medication if deemed necessary for any reason, including pregnancy, intolerable side effects and non-compliance. If acceptable and safe for the participant, cases should be discussed with the Chief Investigator (CI) prior to stopping trial medication. Following cessation of trial IMP, the participant will be managed as deemed clinically appropriate by the investigator/treating clinician. Where possible, participants and investigators will be encouraged to continue the allocated trial activities and follow-up.

#### Strategies to improve adherence to interventions {11c}

Trial medication compliance will be assessed and recorded in the medical records during each trial visit and telephone follow-up. Participants will be asked whether they have had any issues in taking the trial IMP or nIMP and whether any doses have been missed. Compliance issues will be managed on a case-by-case basis by the Principle Investigator (PI).

Participants will be reminded to complete their trial medication diary on a daily basis. At the end of treatment (EoT) visit, the participant diary will be collected, and entries checked against the information recorded in the participant medical records. Participants will be asked to return any unused IMP and nIMP along with all IMP packaging at the EoT visit. Pharmacy will undertake IMP and nIMP accountability.

#### Relevant concomitant care permitted or prohibited during the trial {11d}

No concomitant medications are prohibited; however, statins and fibrates should be used with caution due to an increased risk of musculoskeletal toxicity when nicotinic acid is administered alongside these medications. Participants must not be taking medications that increase the risk of bleeding from the biopsy site at the time of the procedure; they must be able to safely stop any such medication for sufficient time before and after the biopsy to negate the risk.

#### Provisions for post-trial care {30}

Following participation in the trial, participants will return to standard care.

As Sponsor, the Newcastle upon Tyne Hospitals NHS Foundation Trust will provide indemnity in respect of potential liability and negligent harm arising from clinical care. Indemnity in respect of potential liability arising from negligent harm related to trial design is provided by Newcastle University. This is a non-commercial trial and therefore there are no arrangements for non-negligent compensation

### Outcomes {12}

Outcomes are detailed in Table 2 and may be summarised as follows:

**Table 2:** Outcome measures

Primary outcome:	The change in ATP content in skeletal muscle biopsy specimens
Secondary outcomes:	1. Health-related quality of life - The Quality of Life in Neurological Disorders (NeuroQol), evaluates and monitors the physical, mental and social effects experienced by adults and children living with neurological conditions [13] - Newcastle Mitochondrial Quality of life questionnaires (NMQ), psychometric evaluation of mitochondrial disease-specific health-related quality of life [14] - Visual Analogue Scale (VAS) for pain and most bothersome symptom, measuring intensity of symptoms [15] 2. Reported perceived fatigue - Fatigue Impact Scale (FIS) assesses symptoms of fatigue. Evaluates the effect of fatigue on daily life for cognitive, physical and psychosocial functioning [16, 17] - Fatigue Severity Scale (FSS) will be used to measure the impact and severity of fatigue [18] 3. Symptom-limited cardiopulmonary fitness (optional) Performed on a recumbent cycle ergometer with resistance consistently increased via a ramp protocol at 5 to 15 watts/minute. Variables measured at peak (volitional exhaustion) included: - VO_2_, VCO_2_, anaerobic threshold (AT), pulmonary ventilation (VE), respiratory exchange ratio (RER) and breathing frequency (F) - Work rate (power, measured in watts) - Heart rate (HR), stroke volume (SV), cardiac output (Q), and arteriovenous oxygen difference (a-VO_2_ diff) - Rate of perceived exertion [19] - Blood lactate (mmol/L) - Blood oxygen saturation (SpO_2_) (estimated via pulse oximetry) 4. Disease burden - Newcastle Mitochondrial Disease Adult Scale (NMDAS), a semi-quantitative clinical rating scale to assess disease burden for all forms of mitochondrial disease [20] 5. Upper and lower limb function, balance and walking - 6-Minute Walk Test (6MWT) assesses aerobic capacity and endurance, measuring distance covered over 6 min comparing changes in performance [21] - 10-Metre Walk Test (10MWT), assesses walking speed in metres per second over a short distance and can help determine functional mobility, gait and vestibular function [22] - Mini Balance Evaluation Systems Test (Mini-BESTest), to detect balance impairments [23] - 9 Hole Peg Test (9HPT), to measure finger dexterity [24] - 30-second Sit To Stand (STS) to evaluate lower extremity functional strength [25] - Scale for the Assessment and Rating of Ataxia (SARA) assesses different impairments in cerebellar ataxia [26, 27] 6. Skeletal muscle analyses - ATP/ADP ratio via luminescence assay - mtDNA copy number as a marker of mitochondrial density using q-RT PCR. - Respiratory chain deficiency using sequential cytochrome c oxidase and succinate dehydrogenase (COX/SDH) histochemistry and quadruple immunofluorescence - mtDNA heteroplasmy via q-RT PCR or pyro sequencing
Exploratory outcomes	- Investigation of mitochondrial disease markers via Enzyme-Linked Immunosorbent Assay (ELISA) (Fibroblast Growth Factor 21) (FGF-21) and Growth Differentiation Factor 15 (GDF15) - NAD^+^/NADH ratio via luminescence assay - mtDNA heteroplasmy in blood and urine via q-RT PCR or pyro sequencing - Assessment of diabetes markers (insulin, HbA1C, C-peptide and glucose) - Instrumented gait analysis, to assess differences in gait parameters [28] - Assessment of habitual physical activity and sleep using wrist-worn accelerometers worn continuously over 7 consecutive days (optional) - Assessment of participant self-reported symptoms captured via daily Visual Analogue Scales (VAS) [24] for pain and most bothersome symptom and fatigue via Daily-Fatigue Impact Scale (D-FIS) [29] - Skeletal muscle mitochondrial morphology via Electron Microscopy (EM) will be performed for a subset of participants where sufficient tissue is available - Quantitative proteomics analysis, including assessment of PGC-1α and SIRT-1 mediated mitochondrial biogenesis, carried out on a sub-set of samples, should funding and tissue be available.

#### Primary outcome

The primary outcome is the change in energy production (ATP content measured in nmol/mg) in skeletal muscle biopsy specimens between baseline and end of 12 weeks’ treatment.

#### Secondary outcomes

Secondary outcomes include change in measurements of health-related QoL, reported perceived fatigue, disease burden, balance, upper and lower limb function and symptom-limited cardiopulmonary fitness (optional). Skeletal muscle biopsy specimens will be analysed for changes between baseline and EoT in ATP/ADP ratio, NAD^+^/NADH ratio, mtDNA copy number, respiratory chain activity status and mtDNA heteroplasmy.

#### Exploratory outcomes

Exploratory outcomes will include further laboratory analysis of skeletal muscle biopsy specimens to pursue potential disease markers and physiological characterisation of mitochondrial disease (including heteroplasmy and morphology). Other exploratory outcomes will include habitual physical activity, gait analysis and patient-reported symptoms (pain, most bothersome symptom and fatigue).

### Participant timeline {13}

**Fig. 1:**
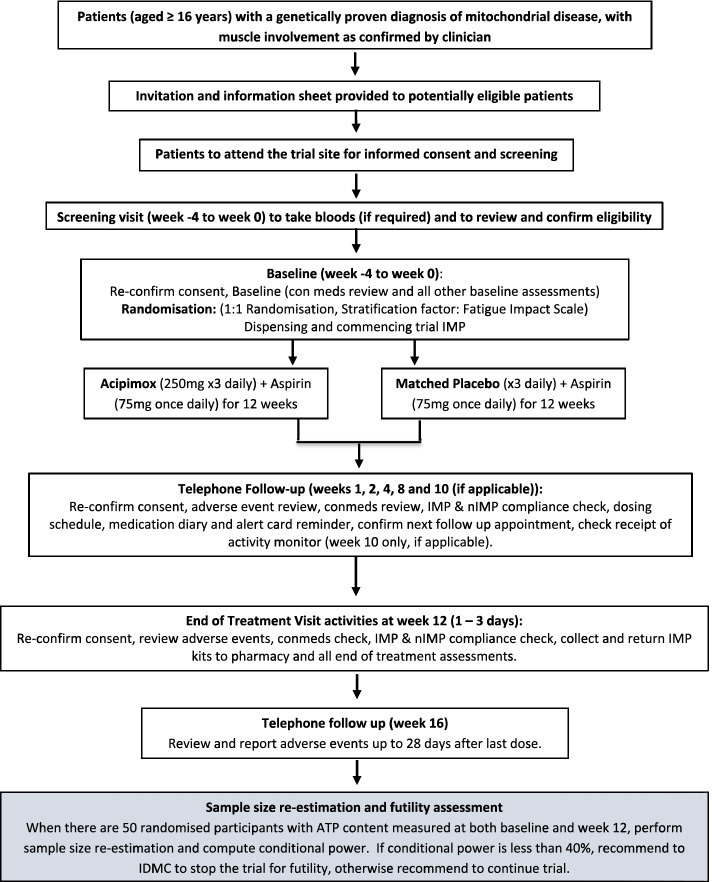
Participant timeline

The trial flowchart for participant timeline is shown in Fig. 1.

#### Participant visits

The AIMM Schedule of Events for participant visits is shown in Table 3.

**Table 3:** Schedule of events

Trial procedure	Day −28 to day 0			Day 1 to day 84	Day 81 to day 90	
	Screen	Baseline		Active treatment period	End of treatment visit (week 12)	End of trial follow-up (week 16)
		Part 1	Part 2	Week 0 to week 12		
Visit Window date	Day −28 to −2^a^	Day −28 to 0^c^		+/7 days	Day 81–90^**f**^	+28 days to +35 days
Informed consent	X					
Demographics	X					
Medical history	X					
Medication review	X	X	X		X	
Eligibility blood samples^b^	X					
Pregnancy test^d^	X					
Contraceptive counselling	X					
Eligibility confirmation	X					
Fatigue Impact Scale			X		X	
Re-confirm Consent		X	X	X	X	
Activity Monitor		X		X^i^		
Physical/Vital signs	X				X	
Urine sample			X		X	
Blood samples^e^			X		X	
Cardiopulmonary exercise test **(Optional)**			X		X	
Functional outcomes			X		X	
NMDAS			X		X	
Questionnaires			X		X	
Needle Muscle biopsy			X		X	
Randomisation^g^			X			
IMP and nIMP dispense			X			
Adverse event reporting				X	X	X
IMP/nIMP Packaging returned					X	
Telephone follow-up^h^				X		

^a^All screening assessments must be completed prior to baseline visit

^b^Eligibility screening bloods will be taken if routine blood results are not available from The Newcastle upon Tyne Hospitals NHS Foundation Trust within the previous 3-month period

^c^All baseline part 1 assessments must be completed prior to all baseline part 2 and randomisation

^d^Females of childbearing potential only

^e^Blood samples: creatinine clearance (U&Es), full blood count (for platelets), liver enzymes (ALT, AST, Gamma GT)

^f^All part 1 assessments must be completed prior to all part 2

^g^Randomisation will only take place once all assessments have been completed

^h^Follow-up phone calls will take place at weeks 1, 2, 4, 8 and 10 (week 10 is only applicable for participants involved in activity monitoring)

^i^An activity monitor will be sent out at week 10 so participants can wear the monitor for the last 7 days prior to the end of treatment visit

### Sample size {14}

A randomised double-blind, placebo-controlled crossover trial of acipimox on mitochondrial function in 21 patients with a diagnosis of type 2 diabetes provided the raw data which has been used in the parallel group design sample size calculation, using established methods [12, 30]. Sufficient muscle samples to measure ATP content were available in nine participants; this resulted in uncertain initial estimates and hence the adoption of an adaptive design with sample size re-estimation. A minimum clinically important difference (MCID) between treatment groups at 12 weeks (follow-up) was considered to be a 1.5-fold increase on average in ATP content in the intervention group compared to the placebo group. The MCID was reached by clinical consensus after consideration of the available published data. This 1.5-fold increase was used to calculate a target absolute difference between treatment groups at 12 weeks for input to the sample size calculation, based on the available pilot data.

### Recruitment {15}

Recruitment approaches have been chosen to optimise participation and mitigate potential unblinding and bias.

#### Clinic appointments

Potentially eligible participants will be identified and approached via the routine NHS Highly Specialised Mitochondrial Service outpatient clinics and associated Multidisciplinary Team Meetings.

#### Wellcome centre for mitochondrial research patient cohort

The Wellcome Centre for Mitochondrial Research Patient Cohort (MitoCohort), previously known as the MRC Mitochondrial Disease Cohort (REC Ref: 13/NE/0326), is a multicentre collaboration with University College London and Oxford University Hospitals and has >1800 registered patients with extensive storage of clinical, biochemical and genetic information. Patients registered on the cohort have consented to receive information about clinical trials they may be eligible for. The cohort database will be interrogated for patients aged ≥ 16 years with mitochondrial myopathy.

#### Advertisement

The trial will be publicised on a number of websites including those of the Wellcome Centre for Mitochondrial Research (www.newcastle-mitochondria.com), mitochondrial disease charities and partner organisations. Copies of the website advert and trial posters may be used in clinical areas. The trial will also be promoted at public and patient facing events and conferences.

#### Participant identification Centres (PICs)

To increase the population covered by the trial, secondary care sites will be set up as PICs to signpost potentially eligible individuals to the recruitment site. Identification of participants will be conducted by the PIC clinical staff. Potential participants will be provided with the PIS and referred to trial site in Newcastle.

#### Minimising risk of bias/unblinding

Due to the inheritability of the disease, eligible patients may be related to each other. To minimise the risk of bias and unblinding, we will avoid simultaneous participation of closely related family members.

## Assignment of interventions: allocation

### Sequence generation {16a}

Randomisation must take place within 28 days of a successful screening visit. The Newcastle Clinical Trials Unit (NCTU) secure web-based randomisation system will be used with variable length random permuted blocks within strata. Randomisation will be stratified by baseline Fatigue Impact Scale (FIS) score (dichotomised as < 40, ≥ 40).

### Concealment mechanism {16b}

See Implementation section {16c} and blinding section {17a}, {17b}. IMP packs generated by the system do not identify whether contents are IMP or placebo.

### Implementation {16c}

Following informed consent, confirmation of eligibility and completion of baseline assessments, trained staff will randomise the participant to one of the two trial arms (acipimox or matched placebo). The system will generate a unique participant trial identifier, a pre-populated prescription, and will allocate an IMP pack number to be dispensed to the participant.

## Assignment of interventions: blinding

### Who will be blinded {17a}

Participants and site staff will not know the treatment allocation assigned to each participant. The Trial Management Group (TMG) will also be blinded to the treatment allocation, with the exception of the Statistician who will perform the interim analysis futility assessment. The Independent Data Monitoring Committee (IDMC) will be partially blinded to the treatment allocation with the closed reports identifying treatment arms as ‘A’ and ‘B’. The Trial Steering Committee (TSC) will remain blinded unless it is deemed necessary to unblind by the IDMC.

### Procedure for unblinding if needed {17b}

Emergency unblinding will only occur for valid medical or safety reasons. It should be carried out by a medically qualified member of the trial team using the randomisation system. In the event of system failure, or absence of such a delegated individual, the treating clinician can request the participant be unblinded by use of sealed code break envelopes held within the site Pharmacy. Details including the participant number, member who broke the code, reason for unblinding and when will be recorded in the Investigator Site File, and Sponsor and the NCTU will be informed at the earliest opportunity. If the event that led to the unblinding meets the criteria for a SUSAR, the standard reporting guidelines will be followed. The PI should not need to be informed what arm the participant was allocated to and the participant will have their trial treatment discontinued. The participant may remain in the trial to be followed up as per protocol. Participants will not routinely be unblinded once they have completed trial treatment.

## Data collection and management

### Plans for assessment and collection of outcomes {18a}

Roles and responsibilities for collection of data, including consent, safety data and outcome measures, will be allocated by the PI and clearly defined in the delegation log. Specific details regarding consent and chosen outcome measures are included above in **{26a}** and **{26b}** and in Table 2. All data collected for an individual participant will be recorded in the trial database (MACRO). Participant identification on the MACRO electronic Case Report Form (eCRF) will be through a unique trial identification number. Participants cannot be identified from eCRFs; records linking participant identity to the unique trial identification number will be stored in the ISF in accordance with the General Data Protection Regulations 2018.

### Plans to promote participant retention and complete follow-up {18b}

All participants choosing to discontinue trial medication should be encouraged to continue with trial follow-up to ensure that information relevant to trial design, medication tolerability and efficacy is collected where possible.

### Data management {19}

Overall responsibility for the quality of data collection and retention lies with the CI. The CI or nominated designee will monitor completeness and integrity of data recording in eCRFs and will correspond regularly with site staff to maintain quality.

Data will be handled, computerised and stored in accordance with the General Data Protection Regulations 2018, and access will be appropriately controlled. All trial data will be retained in accordance with the latest Directive on GCP (2005/28/EC) and local policy.

### Confidentiality {27}

Personal data will be regarded as strictly confidential. All data retained at site and sent electronically to the NCTU will contain the unique trial identification number only. The secure password-protected trial database will also require a date of birth for participant identification and verification, the randomisation system and secure email Serious Adverse Event (SAE) reporting.

A Participant Identification List will be the only document retained within the ISF, which contains full details of hospital number, participant name and unique trial identification number.

### Plans for collection, laboratory evaluation and storage of biological specimens for genetic or molecular analysis in this trial/future use {33}

Trial biological samples (blood, urine, and muscle) will be collected by the PI or appropriately trained designated nominee and analysed either in the Wellcome Centre for Mitochondrial Research Clinical Trials Lab, Newcastle University or The Newcastle upon Tyne Hospitals NHS Foundation Trust Blood Sciences department.

All trial samples are linked to the full trial dataset in anonymous form. Participant NHS number may also be collected and stored within the biobank in order to allow multiple samples from the same donor to be linked.

## Statistical methods

### Statistical methods for primary and secondary outcomes {20a}

Analysis populations are defined as follows:

- *Intention to treat (ITT)*: this population includes all randomised participants regardless of whether they were later found to be ineligible, did not adhere to the protocol or were never treated.
- *Per protocol (PP)*: this population contains all randomised participants who received their allocated trial treatment without major protocol deviations.
- *Safety population*: this population contains all randomised participants who received at least one dose of trial IMP and will be classified according to the actual treatment received.

The analysis of the primary outcome will be in the ITT population with sensitivity analysis in the PP population. The safety population will be used to report side effects.

#### Statistical methods for primary and secondary outcomes

The primary outcome is the change in ATP content in skeletal muscle biopsy specimens, expressed as an absolute difference in nmol/mg of muscle tissue (acipimox minus placebo), between baseline and EoT. The treatment comparison will be made using an analysis of covariance (ANCOVA), with adjustment for baseline ATP content and baseline FIS score. The results will be presented as a 95% confidence interval for the mean difference in ATP content between randomised treatment groups at 12 weeks, adjusted for baseline ATP content and baseline FIS. A two-sided *p*-value of less than 0.05 will be used to indicate statistical significance.

Secondary outcomes will be also be measured at the EoT visit at week 12.

Analyses will follow the same approach as that described for the primary outcome measure. For each secondary outcome measure, the results will be presented as a 95% confidence interval for the mean difference between randomised treatment groups at 12 weeks, adjusted for the baseline of the outcome measure and baseline FIS.

Validated questionnaires and scales will be scored using the scoring algorithm provided. Where a scoring algorithm does not incorporate a method for deriving a total score in the presence of missing questionnaire/scale items, appropriate methods of imputation will be explored.

Analyses of exploratory outcomes will be descriptive; graphical displays and summary statistics will be presented. Where appropriate 95% confidence intervals will be reported.

### Interim analyses {21b}

The interim analysis will include the following:

A sample size re-estimation based on the pre-specified relative effect size of a 1.5-fold increase on average in ATP content in the intervention group compared to the placebo group at week 12. The pooled standard deviation at week 12 will be calculated and will replace the standard deviation used in the original sample size calculation. Although the pre-specified relative effect size will remain fixed, a change in the units of measurement of ATP content from RLU/mg protein (at the time of the sample size calculation) to nmol/mg of muscle tissue necessitates a re-calculation of the absolute mean difference used in the sample size re-estimation (using only the placebo arm data). The sample size may be adjusted upwards to a maximum of 120 participants; it will not be adjusted downwards and so the minimum number of randomised participants will be fixed at a total of 80. This approach to sample size re-estimation has been shown [31] to have a negligible effect on the Type 1 error rate and to preserve the power of the trial.

A futility assessment will utilise the originally hypothesised relative treatment effect size. The conditional power of achieving a statistically significant result at the end of the trial, given the originally hypothesised relative treatment effect, will be calculated [32].

If the conditional power falls below a stopping boundary of 40%, our recommendation to the IDMC will be to stop the trial for futility. Other factors will also be taken into account as part of this decision such as recruitment, adverse events (AEs) and secondary outcome measures data.

### Methods for additional analyses (e.g. subgroup analyses) {20b}

There will be no subgroup analysis, aside from intention to treat (for primary outcome), per protocol (for sensitivity analysis) and safety population (for side effects) analyses. There is further detail on these analysis populations above in ‘Statistical methods for primary and secondary outcomes’ section {20a}.

### Methods in analysis to handle protocol non-adherence and any statistical methods to handle missing data {20c}

As mentioned above, we will analyse both ITT and PP populations. The amount and pattern of missing ATP content data will be examined and multiple imputation techniques, which will allow appropriate estimation of the treatment effect (and associated standard error) whilst utilising data on all participants, may be considered. Otherwise, any participant with missing ATP content data at either the baseline or EoT visit at week 12 will not be included in this analysis.

### Plans to give access to the full protocol, participant-level data and statistical code {31c}

The full protocol and data generated from the research will be available to other researchers once all manuscripts are accepted for publication. The data resource will be identifiable via our publications, presentations at conferences and trial information on a publicly accessible register of clinical trials.

This will comply with the Medical Research Council guidance policy on data sharing and be governed by the principles of the Data Protection Act. For details of the procedure for data sharing, please see the section ‘Availability of data and materials {29}’.

## Oversight and monitoring

### Composition of the coordinating Centre and trial steering committee {5d}

The CI has overall responsibility for the conduct of the trial. The trial is managed by staff based at the NCTU. A TMG, facilitated by the NCTU, will convene approximately monthly throughout the duration of the trial. Members will consist of the CI, key NCTU staff, key local members of the clinical trial project management, delivery and oversight team, trial statisticians, pharmacy, and a Sponsor representative.

A TSC (Trial Steering Committee) and IDMC (Independent Data Management Committee) will be convened to provide oversight of the trial, meeting twice a year. The TSC is composed of clinicians, a statistician and a patient representative. The charter for the TSC is held by the NCTU and may be provided on request.

### Composition of the data monitoring committee, its role and reporting structure {21a}

The data monitoring committee (IDMC) is independent of the sponsor and is composed of senior clinicians and a statistician. Only the IDMC has access to unblinded outcome data before the trial ends; they provide oversight of the safety and integrity of the data. The IDMC reports to the TSC. Their charter is available from NCTU on request.

### Adverse event reporting and harms {22}

Any untoward medical occurrence in a participant following consent and up to 28 days after the last dose of trial IMP will be considered an AE. AEs reported after consent but prior to starting the IMP will be recorded as pre-treatment AEs. Participants who have discontinued trial IMP but who continue in the trial will have AEs collected up to until 28 days after last dose of trial IMP. Events after this date until and including the week 12 visit will only be recorded if related to a trial activity, i.e. muscle biopsy or cycle ergometry.

SAEs occurring following first dose of trial IMP up until 28 days after the last dose will be assessed for causality to the treatment, and expectedness relative to the Reference Safety Information.

### Frequency and plans for auditing trial conduct {23}

The NCTU staff will monitor trial conduct and data integrity to ensure the trial is conducted in accordance with the latest directive on GCP (2005/28/EC). This will be detailed in a Data Management Plan and a Monitoring Plan approved by the trial Sponsor. The main areas of focus will include consent, SAEs, protocol deviations and essential documents. All monitoring findings will be reported and followed up in a timely manner.

The trial may be subject to audit by the Newcastle upon Tyne Hospitals NHS Foundation Trust under their remit as sponsor and inspection by the Medicines and Healthcare products Regulatory Agency (MHRA).

### Plans for communicating important protocol amendments to relevant parties (e.g. trial participants, ethical committees) {25}

Trial Sponsor is responsible for authorising amendments and trial procedures must not be changed without the agreement of the CI, Sponsor, TMG and TSC.

Substantial amendments will be submitted to the appropriate regulatory authorities by the NCTU and will not be implemented until this approval is in place.

Amendments which may impact site will be submitted to the relevant NHS R&D Departments to determine if the amendment affects the NHS permission for that site.

Once approvals are in place, the amendments will be circulated to the wider AIMM team; training will be provided and participants reconsented if necessary.

## Dissemination plans {31a}

We intend this trial will be presented at national and international conferences and published in peer-reviewed journals. Reports will be written for the trial funder, Sponsor and regulatory bodies. The trial will be presented on the Wellcome Centre for Mitochondrial Research website in addition to charity websites including the Lily Foundation.

Once results are available at end of the trial, participants will be contacted directly by letter to thank them for their participation and to provide a summary of the trial results.

## Discussion

As a result of the COVID-19 pandemic and following agreement from the TMG and Sponsor, all AIMM trial activity was suspended on 20 March 2020.

The decision was based on the risk the pandemic posed to the participant population who were considered highly vulnerable. With the uncertainties and escalating national situation around COVID-19, and with NHS research resources being redeployed, the continued follow-up of these participants was envisaged to become increasingly difficult. Participants were therefore withdrawn from the trial, asked to cease trial treatment immediately and followed up as per protocol for 28 days following IMP cessation. The TSC and IDMC were informed and were fully supportive of the suspension.

The situation was monitored closely, and adaptions were made to the protocol in order to mitigate potential risks to the participant population and accommodate the infection control requirements of the Newcastle Hospitals NHS Foundation Trust. This included making the Cardiopulmonary Exercise Testing (CPET) assessment optional. Risk assessment was on-going and once the overall risk was reduced and NHS research resource once again available, screening and recruitment for AIMM re-started.

The suspension of trial activities due to the COVID-19 pandemic impacted on the trial in a number of ways. These included delays to the interim analysis, the stability and viability of the long-term storage of primary outcome muscle biopsy samples, ethical considerations around re-recruiting withdrawn participants and trial IMP reaching the end of its designated shelf life. A funded trial extension has been requested as a consequence of this impact.

## Trial status

The AIMM trial is currently working to protocol version 5.0, dated 3^rd^ June 2021. It is temporarily suspended due to delay in availability of IMP. Recruitment began on 29 August 2019 and is due to end on 30 June 2023.

## Supplementary Information

**Additional file 1.** Informed consent form.

**Additional file 2.** Participant information sheet.

**Additional file 3.** PIS Summary.

## Data Availability

The CI and collaborators will act as custodians of the trial data and manage data sharing requests through ReShare, a UK online data repository recommended by funder. Parties interested in data sharing will directly contact the CI and collaborators and complete a project proforma and provide the rationale, the information required and to comply with the regulation of responsibilities of users. If agreed to be appropriate, requested data will be made available. An access advisor who is independent of the trial team will periodically review access decisions.

## Abbreviations

ADP: Adenosine diphosphate
AE: Adverse event
AT: Anaerobic threshold
ATP: Adenosine triphosphate
a-vO_2_ diff: Arteriovenous oxygen difference
CI: Chief Investigator
COX: Cytochrome *c* oxidase
CPET: Cardiopulmonary Exercise Testing
eCRF: Electronic Case Report Form
eGFR: Estimated Glomerular Filtration Rate
ELISA: Enzyme-linked immunosorbent assay
EM: Electron microscopy
EudraCT: European Clinical Trials Database
F: Breathing frequency
FGF-21: Fibroblast growth factor 21
FIS: Fatigue Impact Scale
FSS: Fatigue Severity Scale
GCP: Good Clinical Practice
GDF15: Growth differentiation factor 15
GDPR: General Data Protection Regulation
9HPT: 9 Hole Peg Test
IDMC: Independent Data Monitoring Committee
IMP: Investigational Medicinal Product
ISRCTN: International Standard Randomised Controlled Trials Number
MHRA: Medicines and Healthcare products Regulatory Agency
Mini-BESTest: Mini Balance Evaluation Systems Test
6MWT: 6-Minute Walk Test
10MWT: 10-metre Walk Test
NAD^+^: Nicotinamide adenine dinucleotide (oxidised)
NADH: Nicotinamide adenine dinucleotide (reduced)
NCTU: Newcastle Clinical Trials Unit
nDNA: Nuclear DNA
NHS: National Health Service
nIMP: Non-Investigational Medicinal Product
NMDAS: Newcastle Mitochondrial Disease Adult Scale
OXPHOS: Oxidative phosphorylation
PGC1α: Peroxisome proliferator-activated receptor-gamma coactivator
PICS: Participant Identification Centres
PIS: Participant Information Sheet
PPIS: Pregnant Partner Information Sheet
Q: Cardiac Output
q-RT PCR: Quantitative Reverse Transcription PCR
REC: Research Ethics Committee
RER: Respiratory Exchange Ratio
SAE: Serious Adverse Event
SARA: Scale for the Assessment and Rating of Ataxia
SDH: Succinate dehydrogenase
SIRT-1: Sirtuin 1
STS: 30-second Sit To Stand
SV: Stroke volume
TMG: Trial Management Group
TSC: Trial Steering Committee
VAS: Visual Analogue Scale
VE: Pulmonary ventilation
