# Correction: Acipimox in Mitochondrial Myopathy (AIMM): study protocol for a randomised, double-blinded, placebo-controlled, adaptive design trial of the efficacy of acipimox in adult patients with mitochondrial myopathy

**DOI:** 10.1186/s13063-022-06814-8

**Published:** 2022-10-05

**Authors:** The AIMM Trial Group:, Alaa Abouhajar, Lisa Alcock, Theophile Bigirumurame, Penny Bradley, Laura Brown, Ian Campbell, Silvia Del Din, Julie Faitg, Gavin Falkous, Grainne S. Gorman, Rachel Lakey, Robert McFarland, Jane Newman, Lynn Rochester, Vicky Ryan, Hesther Smith, Alison Steel, Renae J. Stefanetti, Huizhong Su, Robert W. Taylor, Naomi J. P. Thomas, Helen Tuppen, Amy E. Vincent, Charlotte Warren, Gillian Watson

**Affiliations:** 1 Newcastle Clinical Trials Unit, 1-4 Claremont Terrace, Newcastle University, Newcastle upon Tyne, NE2 4AE UK; 2 Brain and Movement Research Group, Clinical Ageing Research Unit, Translational and Clinical Research Institute, Newcastle University, Newcastle upon Tyne, NE4 5PL UK; 3 Population Health Sciences Institute, Newcastle University, Ridley 1 Building, Newcastle upon Tyne, NE1 7RU UK; 4 Pharmacy Directorate, Newcastle Upon Tyne Hospitals NHS Foundation Trust, Freeman Hospital, Freeman Road, Newcastle Upon Tyne, NE7 7DN UK; 5 Wellcome Centre for Mitochondrial Research, Translational and Clinical Research Institute, Faculty of Medical Sciences, Newcastle University, Newcastle Upon Tyne, NE2 4HH UK; 6 NHS Highly Specialised Service for Rare Mitochondrial Disorders, Newcastle Upon Tyne Hospitals NHS Foundation Trust, Newcastle Upon Tyne, NE1 4LP UK

**Correction: Trials 23, 789 (2022)**

**https://doi.org/10.1186/s13063-022-06544-x**

Following the publication of the original article [1], we were notified that the name of the 7^th^ author has been incorrectly spelled.

Originally published name: Sylvia Del Din

Corrected name: Silvia Del Din

The original article has been corrected.
