# Supplementary material for: Acipimox in Mitochondrial Myopathy (AIMM): study protocol for a randomised, double-blinded, placebo-controlled, adaptive design trial of the efficacy of acipimox in adult patients with mitochondrial myopathy
Source: Trials. 2022 Sep 20;23:789. doi: 10.1186/s13063-022-06544-x (PMC9486776; doi:10.1186/s13063-022-06544-x)
Supplement: Supplementary file 1 — Additional file 1. Informed consent form. [file 13063_2022_6544_MOESM1_ESM.docx]

**Chief Investigator:** Professor Gràinne Gorman, Mitochondrial Research Group, Newcastle University, NE2 4HH

**Sponsor:** The Newcastle Upon Tyne Hospitals NHS Foundation Trust

**AIMM: Acipimox in Mitochondrial Myopathy**

**Informed Consent Form**

**Please INITIAL the box where you agree. Please note that you do not need to agree to every statement:**

| 1. | I confirm that I have read and understand the information sheet dated ……………………… (version …………..) for the above trial. I have had the opportunity to consider the information, discuss the risks and inconveniences, ask questions and have had these answered satisfactorily. | | |  |
| --- | --- | --- | --- | --- |
| 2. | I agree to my General Practitioner and other relevant healthcare professionals being informed of my participation in the trial, including any necessary exchange of information about me between my GP, other relevant healthcare professional and the research team. | | |  |
| 3. | I understand that my participation in the trial is entirely voluntary and that I am free to withdraw at any time, without giving any reason and without affecting my routine or future medical treatment. | | |  |
| 4. | I agree to take the trial medication as prescribed, attend the required trial visits and follow advice provided to ensure my safety as outlined in the patient information sheet. I understand that if I do not engage with these safety measures that I will be unable to continue in the trial. | | |  |
| 5. | I understand that a copy of this consent form will be submitted to Newcastle Clinical Trials Unit for the purposes of central monitoring, and will be destroyed following a documented check of the form. I give permission for these individuals to receive a copy of this form. | | |  |
| 6. | I understand that relevant sections of my medical notes and data collected during the trial may be looked at by individuals from regulatory authorities, from the NHS Trust, or from Newcastle University where it is relevant to my taking part in this research. I give permission for these individuals to have access. | | |  |
| 7. | I understand that the information collected about me during this trial will be used to support other research in the future, and may be shared anonymously with other researchers. | | |  |
| 8. | I agree to take part in the above trial. | | |  |
| 10. | I agree that any samples I have given (blood, urine and skeletal muscle) can be stored anonymously in the Newcastle Mitochondrial Research Biobank (REC Ref: 16/NE/0267). These samples may be used in approved research projects.  If you **agree** for samples to be kept for use in the future, please also answer the following questions: | | |  |
|  | | Do you give permission for your samples to be used in research projects that use animals. | **YES NO** | |
|  | | Do you give permission for your samples be used in commercial research projects. | **YES NO** | |

____________________ _________________ _____________________

Name of Participant Date (dd/mm/yyyy) Signature

(Please print)

____________________ _________________ ______________________

| Name of Doctor taking Consent(Please print) | Date (dd/mm/yyyy) | Signature |
| --- | --- | --- |

If the patient is able to provide informed consent but unable to sign this consent form due weakness or ataxia as a result of their condition, consent should be confirmed orally in the presence of a witness

_________________________________

Name of participant

________________________ ___________ _____________________

Name of witness Date Signature

When completed: 🞏 original to site file 🞏 1 copy to medical notes 🞏 1 copy to participant 🞏 Copy to Clinical Trials Unit
