# Supplementary material for: Acipimox in Mitochondrial Myopathy (AIMM): study protocol for a randomised, double-blinded, placebo-controlled, adaptive design trial of the efficacy of acipimox in adult patients with mitochondrial myopathy
Source: Trials. 2022 Sep 20;23:789. doi: 10.1186/s13063-022-06544-x (PMC9486776; doi:10.1186/s13063-022-06544-x)
Supplement: Supplementary file 2 — Additional file 2. Participant information sheet. [file 13063_2022_6544_MOESM2_ESM.docx]

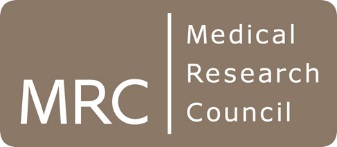

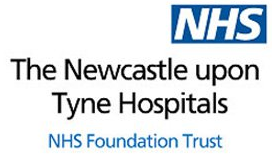


**
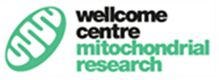
** **
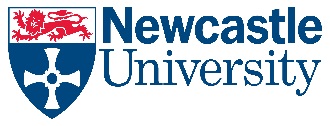
**

**AIMM: Acipimox in Mitochondrial Myopathy**

**Patient Information Sheet**

You have been invited to take part in the AIMM Clinical Trial.

You should have already received and read the Summary Patient Information Sheet and decided that you would like to find out more about taking part in the trial.

This Patient Information Sheet contains further details about the clinical trial including why you have been invited to participate, what taking part will involve for you and who to contact if you have any questions or concerns.

This information sheet is divided into sections.

Before you make a decision on whether to take part, please make sure you read this information sheet **carefully and** **in full**.

Please ask if anything is not clear.

# **Summary**

Muscle symptoms including muscle weakness, fatigue, and pain are extremely common and often debilitating in patients with mitochondrial myopathy. To date, there are no licensed treatments and no cures. Hence, there is an urgent need to find an effective drug treatment.

We plan to test a medication (acipimox) that has been used to treat high cholesterol and improve diabetes. This medication has also been shown to boost ATP (energy) levels within muscle cells. It is this function, which may be beneficial to patients with mitochondrial disease with muscle involvement as it may relieve the debilitating muscle symptoms.

The benefits of using a medication that has already been used in other diseases, are that we already understand the effect of the medication on humans, its side effects, and how best to administer it. We also have some understanding about effective doses. This potentially provides a faster and less expensive pathway to address the urgent need for new drug treatments for mitochondrial diseases.

This trial has been designed with the help and support of patients and their care providers. This partnership, which has allowed us to co-design this trial from the start, will continue until the end of the trial.

Patients/their carers were asked about aspects of their disease that they hoped could be addressed with any new treatments. Muscle symptoms were considered to be the most important target. We then worked together to explore the best way to measure aspects of everyday functioning that could benefit from a new drug treatment. For the first time in a clinical trial, we have used an 'adaptive design model', which will allow us to modify the number of participants needed in the trial, as more information is collected.

Involvement in this trial will take place over 16 weeks and participants will take medication three times a day for 12 weeks. Some participants will receive acipimox and some will receive an identical 'placebo' treatment, this is a tablet that looks identical to the acipimox tablet but does not contain any active medication. Neither the doctor nor the participant will know who receives which treatment (double-blind). This is important to ensure that any effects are due to the medication and not to any other potential factors (e.g. more interaction with medical staff or hospital visits).

In addition to taking a small piece of muscle at both the beginning and end of the trial (to better understand the effect of drug and mitochondrial disease on muscle), we will monitor the safety of all participants. We will also perform assessments of everyday functioning including walking, heart and lung capacity, muscle strength and performance, mental agility and the impact of disease symptoms on mental health and well-being.

# **Why are we inviting you to take part?**

You are being invited to take part in this clinical trial because you have had a genetic test that shows you have mitochondrial disease with muscle weakness (myopathy). Myopathy is a common symptom of mitochondrial disease.

You may have previously given consent for your personal information to be stored as part of the Mitochondrial Patient Cohort, and for us to contact you about taking part in research studies. Alternatively, you may have been approached by staff at your local hospital to take part or you may have heard about the trial and contacted the trial team to find out more information.

# **What is this trial going to do?**

**Background**

Mitochondria are the energy-producing units, or batteries, of your cells. If your mitochondria don’t work properly, then areas of your body that require lots of energy often don’t work properly, e.g. brain, heart, muscles.

The type of energy that mitochondria produce in cells is called ATP.

A previous trial has shown that the commonly prescribed medication acipimox increased ATP levels (energy) in the muscle cells of patients with diabetes.

**This Trial**

This clinical trial has been set up to test whether acipimox has an effect on the way your muscle makes energy and whether it is effective in treating your muscle symptoms.

We would like to find out if taking acipimox tablets increases the energy content (ATP/ADP) in the skeletal muscle of people like you with mitochondrial disease.

If you agree to take part in this trial you will be asked to take acipimox or placebo three times a day for 12 weeks. We will also ask you to take a low dose (75mg) of aspirin once every day.

# **Who is organising and funding the trial?**

The trial is being organised by doctors from the Wellcome Centre for Mitochondrial Research at Newcastle University. The doctor in charge of the trial (the Chief Investigator) is Professor Gráinne S Gorman.

The research is being funded by the Medical Research Council, UK.

The Newcastle upon Tyne Hospitals NHS Foundation Trust is the trial sponsor. This means they are responsible for the conduct of the trial.

Your doctor will not be paid for including you in this clinical trial.

The trial will inform part of a PhD study.

# **Will I definitely receive the trial medication, acipimox?**

We will compare acipimox with a placebo tablet. This is a tablet that looks identical to the acipimox tablet but does not contain any active medication. Using a placebo makes it easier for us to determine whether any changes seen in patients who take part in the trial are due to the acipimox or are due to something else. As this is a placebo-controlled trial it is also randomised and double-blinded.

Randomised means that you will be allocated to one of two treatments (acipimox or placebo). In this trial there is an equal chance of you receiving either treatment. Neither you nor the trial doctor or nurse have any control over which treatment you receive and neither you nor the trial doctors or nurses will know whether you are receiving acipimox or placebo.

However, for safety reasons, we can quickly find out which type of tablet you have taken if we need to.

It has been found that taking a low dose of aspirin reduces the chance of experiencing certain mild side effects associated with acipimox. We will therefore ask every patient to take one low-dose aspirin tablet every day while on the trial.

# **Who can take part?**

To take part in this trial, you must:

- Be aged 16 or over.
- Be able to give informed consent to participate in the trial.
- Have a confirmed genetic diagnosis of mitochondrial disease.
- Have evidence of myopathy as determined by a doctor.
- Be on a stable dose of any current regular medication for at least 4 weeks prior to trial entry.
- Not already be taking acipimox.
- Have normal kidney function, with a creatinine clearance of ≥60ml/minute.
- Have blood tests within a normal range.
- Women of child bearing potential and men whose partner is of child bearing potential must be willing to ensure that they, and/or their partner, use effective contraception during the trial and for 28 days thereafter.
- Be willing to take the trial drug (tablets) 3 times every day and to also take aspirin once a day, for 12 weeks.
- Be able to attend both a baseline visit and then another visit after 12 weeks to complete the trial tests. Each trial visit which will last 1-3 days.
- Be willing and able in the doctor’s opinion to comply with all trial requirements.
- Be willing to allow your GP and other health professionals, if appropriate, to be notified of your participation in the trial.

You may not be able to take part in the trial if you have certain health conditions, which mean that you would be unsuitable to receive trial medication or complete the trial assessments. However, we will collect information on your medical history and will do a number of checks to confirm that you are suitable to take part (we call this a screening visit) before we enter you into the trial.

# **Do I have to take part?**

It is up to you to decide whether you want to join the trial. If you agree to take part, we will then ask you to complete a consent form. This will not affect the current or future routine medical care you receive.

# **I’m interested in taking part, what happens next?**

Once you have read this sheet thoroughly and have had an opportunity to discuss it with your friends, family or your GP, a member of the research team will phone you. You will be asked to agree a suitable date to attend a screening visit at the trial centre in Newcastle.

# **What will giving consent mean for me?**

By signing a consent form, this means that you fully understand what taking part in the trial means for you. That’s why it is really important that you can take as much time as you want to read this information sheet and ask lots of questions – especially if there’s something that you don’t understand or are worried about.

All questions are good questions! If you would prefer you can use a pen or highlighter to mark the areas of this sheet that you would like to talk about, and then show it to the doctor.

The consent form lists all the things that you agree to do, or agree for the trial team to do. We cannot do anything that you do not agree with.

Should you be unable to give written consent due to a symptom of your mitochondrial disease, we will ask you to give verbal consent in the presence of an independent witness.

# **What happens if I don’t want to take part anymore?**

You can change your mind about taking part in the trial at any point. You do not have to provide a reason, although this may be helpful for us to understand why you are withdrawing. If you decide to discontinue your trial medication before the end of treatment, we would ask if you would be willing to continue in the trial for follow up and have a second muscle biopsy, and/or cycle test and/or complete a questionnaire before leaving the trial. We will ask you to sign a form agreeing to remain in the trial for follow up.

You are free to withdraw from the trial at any time. We will ask you to sign a form saying that you don’t want to take part anymore. Withdrawing from the trial will have no effect on your normal routine care.

# **What will taking part involve?**

We will invite you to attend 3 visits in Newcastle unless Screening and Baseline visit can be combined as one visit:

- Screening visit (1 day)
- Baseline visit (over 1-3 days)
- End of treatment visit (1-3 days)

These visits are extra visits and not part of your normal care.

At each visit, we will provide, or pay for, transport to the Newcastle clinic and back home again and we will also pay for overnight accommodation if you are travelling from outside the local area. We will provide you with lunch and refreshments throughout the day. Breaks will be scheduled as required.

**Screening Visit**

At the screening visit we will fully discuss the trial with you, and make sure that you understand what taking part in the trial will involve.

After you have given consent, we will review your routine blood test results to make sure it is safe for you to take part in the trial, if you have not had a routine blood test at the hospital in Newcastle within the previous 3 months a blood sample will be taken to confirm your eligibility. Some basic demographic data (Gender, date of birth, ethnicity, smoking status and alcohol intake) will also be collected.

We will also discuss how you, or your partner, should avoid getting pregnant during this trial. We will ask women of child-bearing potential to take a pregnancy test.

If all of the tests indicate that you are suitable for the trial, we will invite you to return for the baseline visit.

**TOTAL TIME FOR SCREENING VISIT IS 3-4 HOURS.**

**SCREENING
& PRE-TREATMENT**

**Screening Visit (up to Week -4 to Week 0):**

- Informed consent
- Medical history and medication review
- Urine pregnancy test (females of childbearing potential)
- Review blood results
- Contraceptive counselling

**Baseline Visit (Week 0):**

- Confirm consent to continue participation
- Activity Monitor dispensing & instructions (if applicable)
- Medical history and medication review
- Physical examination and vital signs
- Urine and blood tests
- Exercise test with ECG and blood test (if applicable)
- Functional assessments of upper and lower limb function balance and walking
- Questionnaires (if not completed before your visit)
- Muscle biopsy
- Receive study treatment

**BASELINE**

**Follow Up telephone call (Week 1, 2, 4 and 8):**

- Review of Informed consent
- Complete diary to record medication taken and pain, fatigue and most bothersome symptom
- Check for adverse events
- Check telephone contact details

**Week 10:** Activity Monitor dispensing & instructions (if applicable)

**ACTIVE TREATMENT**

**TR**

**End of Treatment Visit (Week 12):**

- Review of informed consent
- Physical examination and vital signs
- Urine and blood tests
- Exercise test with ECG and blood tests
- Functional assessments of upper and lower limb function balance and walking
- Questionnaires
- Muscle biopsy

**Week 16:** Follow up telephone contact to check for adverse events

**FOLLOW-UP**

**Baseline and End of Treatment Visits**

This will last 1 - 3 days. You can go back home (or back to your hotel if you are travelling from further afield) at the end of every day. At the end of the baseline visit, we will give you the trial medication (tablets) to take home with you.

During the visit we will ask you if we can perform the following activities with you:

- Perform a physical examination and vital signs (including ECG, height, weight, hip and waist measurements, blood pressure and heart rate)
- Measure how well you walk and move
- Assess your heart and lung function, blood pressure and heart rate by asking you to cycle on a stationary bike. Blood samples will be taken from your ear or arm during the test.
- Obtain a urine sample – we will ask you to fill a small pot in private.
- Perform some blood tests – we will collect about 5 teaspoons of blood each time. One of these blood tests will be a fasted sample. This means that we will ask you not to eat anything for 12 hours prior to the blood sample (you will be able to drink water during this time). We will ask you to come into the trial centre on the morning of the sample without having had breakfast; we will collect your blood sample and will then give you breakfast.
- Obtain a muscle sample – this is a safe, routine clinical procedure where a small piece of muscle, about the size of two small peas is sampled. Usually the muscle is from the front of the thigh but occasionally other sites may be used. We first inject local anaesthetic with a small needle into the skin to numb the area. This usually only causes a mild stinging sensation. However, for some people, needles may cause them to faint. Although most people do not find the procedure to be painful, some people do find the removal of muscle tissue painful, even with the anaesthetic. Each biopsy will leave a small scar about 1cm long.
- Complete some questionnaires - We will ask you to fill in a number of questionnaires about your symptoms. The questionnaires will be sent to you before your screening visit; we will ask first if you are happy for us to do this.
- You may be asked to wear an activity monitor and record the time you wake up and go to be each day in a paper diary

After your End of Treatment visit, you will return to your usual care.

**TOTAL TIME FOR THE BASELINE OR END OF TREATMENT VISIT IS 1 - 3 DAYS, WITH 6 HOURS PER DAY**

# **Why do I have to avoid getting pregnant?**

We don’t know if the trial drug will harm an unborn baby. To be safe, we will discuss with you how to avoid getting pregnant, or avoid your partner getting pregnant.

It is also possible that if acipimox is given to either a man or woman who goes on to conceive a child that it may harm their unborn child. Therefore, women or men with female partners, planning to become pregnant during the course of the trial will be excluded.

If you are a woman of child bearing potential, you will be asked to take a pregnancy test at the baseline visit, and you will not be allowed to take part in the trial if the pregnancy test is positive.

To prevent pregnancy during the trial, all participants of child bearing potential have to use contraception from the day that written consent is given to take part in the trial up until 28 days after the last dose of trial medication is taken.

For male participants contraceptive methods include:

• condom

• practice true abstinence in line with preferred and usual lifestyle

For female participants contraceptive methods include:

• combined hormonal contraception (oral, intravaginal, transdermal)

• progestogen only hormonal contraception (oral, injectable, implantable)

• intrauterine device (IUD)

• intrauterine hormone-releasing system (IUS)

• vasectomised partner

• bilateral tubal occlusion

• practice true abstinence in line with preferred and usual lifestyle

If you or your partner become pregnant during the course of the trial, you must tell your trial doctor immediately so appropriate action can be discussed.

# **What happens if I, or my partner gets pregnant during the trial?**

Any person who finds out that they, or their partner, have become pregnant while taking part in the trial should immediately contact a member of our research team.

If you do become pregnant during the trial, you will need to discontinue trial medication. We will ask you to return your trial medication.

We will monitor your pregnancy carefully and will ask if we can collect some information on the health of your baby when it is born. A children’s doctor will also be asked to check your baby carefully when she/he is born.

If your partner becomes pregnant during the trial, we will ask them to sign a consent form to allow the trial team to collect safety information about their pregnancy and their baby.

# **What happens if I’ve had a baby, but I’m breastfeeding?**

If you are a woman and breastfeeding, you will not be allowed to take part in the trial. This is because we don’t know if the trial medication is passed into breastmilk or if it is safe in babies.

# **Will my GP know that I’m taking part in the trial?**

Yes, with your consent, we will send a letter to your GP to inform them that you are taking part in this trial. We will also inform your mitochondrial consultant or other relevant healthcare professionals.

This is so that your medical records at your GP practice and in hospital have a record that you took part in a clinical trial. Any test results from taking part in this trial will also be added to your hospital medical records.

Neither your GP nor mitochondrial consultant will know if you have received acipimox or placebo.

# **What will happen to my blood, urine and muscle samples?**

We will use some of your samples in tests for this trial, this includes genetic analysis. If there are any samples left over at the end of the trial, we would like to store them in the Newcastle Mitochondrial Research Biobank (16/NE/0267). We would like to do this so that we can use them again in future studies. Samples may only be used by other researchers and research projects that have been subject to proper scientific and ethical review.

We will ask for your consent to store your samples in this way and will also ask if you consent for your remaining samples to be used in research in animals and for commercial research.

If you choose not to donate your remaining samples to the biobank you will still be able to take part in the trial and any samples remaining at the end of the trial will be destroyed.

# **Will my information be kept confidential?**

Yes, all of the information collected in the trial will be entered on computers that are kept secure and password protected.

• You will be given a unique trial identification number instead of your name being written on trial documents. Only the trial team at your hospital will be able to link this number back to you using your date of birth, name and NHS number.

• The trial team at your hospital will have access to your information during the trial to organise trial visits as well as for ongoing safety.

• You will not be named in any results, reports or on websites.

• Very occasionally, information might be given during the trial that we would have a legal obligation to pass on to others (for instance information which suggested you or others were at risk of harm). In this case, confidentiality would be broken so that we could pass this information to the relevant people. You would be informed of this.

• At the end of the trial, all trial information will be kept in a secure storage area (this is called archiving) for at least 5 years. This makes sure any queries about the running of the trial have been answered. All information will be held securely to make sure we protect your confidentiality, after which it will be safely destroyed.

• If there are any serious adverse events, we would send details of this event to the government medicines agency (MHRA). There is a specific form to do this and no personal identifiable data will be transferred, only your trial number will be sent to them.

• Some parts of your medical records and the data collected for the trial may be looked at by authorised persons from the MHRA, sponsor (Newcastle Hospitals NHS Foundation Trust) and or the Newcastle Clinical Trials Unit to check that the trial is being conducted to the correct standards. All will have a duty of confidentiality to you as a research participant.

# **Are there any benefits in taking part in this trial?**

This is the first trial in the world of acipimox in patients with mitochondrial disease, so we do not know whether this trial will help you feel better.

We hope that the information that we collect from this trial will help us understand what drugs may be useful in mitochondrial disease. The results may also be useful in understanding other diseases where muscle tissue is lost or not working properly.

We also hope that the results will help us understand the movement and exercise tests better, so that we can improve our testing techniques in the mitochondrial clinic.

# **Are there any disadvantages in taking part in this trial?**

Trial visits will take between 1 - 3 days. We will pay for your travel to the Newcastle trial centre, parking costs and accommodation (if required). We will also ensure that you are given lunch and refreshments during the visits. Taking part in this trial will not result in any cost to you other than your time.

**Trial drugs**

The trial medications are commonly used and are safe. However, everyone reacts differently to medications and side effects will vary from person to person. The commonly known side effects are:

Acipimox might cause indigestion, headaches, reddening of the skin and hives.

Aspirin can cause increased bleeding and indigestion when given in high doses. However, in this trial, we will ask you to take a very low dose, and this is unlikely to have any side effects.

**Trial Tests**

You will have samples of blood taken. This may sting, but you can ask for some numbing cream or spray. We are only taking the same as 5 teaspoons of blood each time. Having a blood test may leave you with a bruise.

All of the trial tests are safe and routinely used in the mitochondrial clinics. You may feel tired during the tests, but you can ask to stop the test or take a break at any time.

You will have a muscle sample (biopsy) taken at both the baseline and end of treatment visit. A local anaesthetic will be injected first to numb a small area, and this may cause a mild stinging sensation. However, for some people, needles may cause them to faint. Another needle will be used to take the muscle biopsy. You will then have a Wound Closure Strip placed on the area to close the incision. Although most people do not find the procedure to be painful, some people do find the removal of muscle tissue painful, even with the anaesthetic. Each biopsy will leave a small scar about 1cm long.

All the staff involved in performing the various tests with you are specifically trained to do so.

If we find any abnormal results, you will be referred to the clinical team responsible for your care. We would like to inform your GP and mitochondrial specialist, and will ask your permission to do this.

**Patient Diary and Follow up Phone Calls**

You will need to take the trial medication 3 times per day, and aspirin once per day, for 12 weeks in total. The research team can help you to sort out a way to help you to remember to take the tablets.

Every day we will ask you to complete a diary, which will take up some of your time. In the diary we will ask you to record that you have taken your trial medication and aspirin. We will also ask you to record your daily levels of pain, fatigue and most bothersome symptom.

You will have to be available to take a follow up phone call from the research team at weeks 1, 2, 4, 8 and 10 (if applicable). This call will take about 20 minutes and you be asked if you have had any adverse events since your previous visit or call. The call will be arranged for a time that suits you. We will also call you 4 weeks after your End of Treatment visit to check how you have been getting on since finishing the trial medication.

# **What will happen at the end of this trial?**

At the end of the trial (once all participants have completed all assessments), we will write to you to inform you of the trial results.

We will also publish the results of the whole trial in our Newcastle ‘Mitonews’ newsletter, as well as media and peer reviewed journals.

The results will also be available at specialised mitochondrial clinics and on the Wellcome Centre for Mitochondrial Research website (www.newcastle-mitochondria.com). No patients will be identifiable in any of the trial publications.

# **What if something goes wrong?**

We do not expect anything to go wrong as a result of you taking part in this clinical trial. If you have been harmed by taking part in this trial, due to someone’s negligence, you may have grounds for legal action and could seek compensation through the research sponsor, The Newcastle upon Tyne Hospitals NHS Foundation Trust, who have appropriate insurance-related arrangements in place. However, you may have to pay for it. If the harm is due to routine clinical treatment or negligence, then the NHS indemnity arrangements will apply. Regardless of this, if you wish to complain, or have any concerns about any aspect of the way you have been approached or treated during the course of this trial, the normal National Health Service complaints mechanisms may be available to you. Please ask a member of the trial team or see details below.

# **Who has reviewed this trial?**

Mitochondrial patients and their families helped to design this trial. They have also looked at this information sheet, the consent form and patient diary. This trial has also been rigorously reviewed by external experts during the application process for funding, awarded by the Medical Research Council, UK.

The Newcastle Upon Tyne Hospitals NHS Foundation Trust have reviewed all of the trial documentation, and assessed the risks of this trial. They are happy to take responsibility (as sponsor) for this trial.

All clinical trials taking place in the NHS must be reviewed by the Health Research Authority and a Research Ethics Committee. They ensure that we are not doing anything harmful to you during this trial and that your data are collected safely and stored securely.

All NHS clinical trials involving drugs must be reviewed by the Medicines and Healthcare products Regulatory Agency (MHRA).

Both the MHRA and the ethics committee (Research Ethics Committee Northern Ireland) are happy for us to go ahead with the trial.

# **What if I have any concerns?**

If you have any concerns about this trial, please contact Research Nurse

(<insert name>) on **0191 2XXXXXXX** ([@newcastle.ac.uk](mailto:Catherine.feeney@newcastle.ac.uk)).

You can also speak to the trial Chief Investigator, Professor Gráinne Gorman on **0191 282 0340**.

You can also talk to Newcastle Upon Tyne Hospitals Patient Advice and Liaison Service (PALS) by telephoning 0800 0320202.

If you remain unhappy and wish to complain formally, you may use the normal NHS complaints procedure. Details of this procedure can be obtained from the following website:[*http://www.nhs.uk/choiceintheNHS/Rightsandpledges/complaints/Pages/NHScomplaints.aspx*](http://www.nhs.uk/choiceintheNHS/Rightsandpledges/complaints/Pages/NHScomplaints.aspx)

**Thank you for reading this information sheet.**
