# Supplementary material for: Acipimox in Mitochondrial Myopathy (AIMM): study protocol for a randomised, double-blinded, placebo-controlled, adaptive design trial of the efficacy of acipimox in adult patients with mitochondrial myopathy
Source: Trials. 2022 Sep 20;23:789. doi: 10.1186/s13063-022-06544-x (PMC9486776; doi:10.1186/s13063-022-06544-x)
Supplement: Supplementary file 3 — Additional file 3. PIS Summary. [file 13063_2022_6544_MOESM3_ESM.docx]

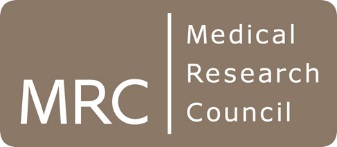

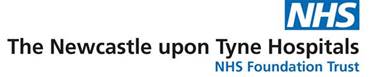


**
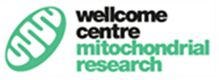
** **
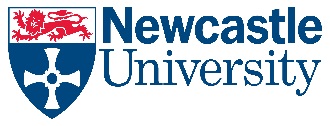
**

**AIMM: Acipimox in Mitochondrial Myopathy**

**Summary Patient Information Sheet**

- We would like to invite you to take part in a clinical trial.
- Before you decide whether or not to take part it is important that you understand why we are doing this research and what it will involve for you.
- On the next page is a brief summary of the clinical trial and what it will involve. This is followed by a trial timeline on page 3. If after reading this summary you are interested in taking part, please read the full Patient Information Sheet which contains more details.
- Please take time to read this information through carefully. Feel free to discuss it with your friends, family or your GP.
- You are free to decide whether or not to take part in this clinical trial. If you decide not to take part it will not affect the treatment you receive from your doctors.
- Please ask if you have any questions or if anything is unclear.

**About the AIMM Trial**

- The AIMM trial is for patients with mitochondrial disease and muscle weakness (myopathy) or exercise intolerance.
- This trial will test whether a drug called acipimox has an effect on the way your muscle makes energy.
- If you take part you will be asked to take acipimox or placebo (a tablet that looks like acipimox but contains no active ingredients) three times a day for 12 weeks. You will also be asked to take a low dose (75mg) of aspirin once every day.
- Taking part will also require you to attend the trial centre in Newcastle for three visits (screening, baseline and end of treatment). You will also receive follow up telephone calls from the research team at weeks 1, 2, 4, 8, (week 10 if applicable) and week 16 to check on your progress and document if you have suffered any adverse events during this time.
- Each visit will last 1-3 days. During these visits we will perform a number of tests (including blood tests and a muscle biopsy) and will ask you to complete a number of activities (cycling on a stationary bike, and how well you walk and move). We will also ask you to complete a number of questionnaires.
- The cost of travel to and from Newcastle, accommodation (for patients who are travelling from outside the Newcastle area) and refreshments will be provided/refunded.

**AIMM Trial Timeline**

**Start of Treatment**

Baseline Visit in Newcastle (over 1-3 days)

Trial Treatment Period

Telephone Follow-ups at week 1, 2, 4, 8 and 10 (if applicable)

**End of Treatment**

End of Treatment Visit in Newcastle (over 1-3 days)

Week 1

Week 2

Week 3

Week 4

Week 5

Week 6

Week 7

Week 8

Week 9

Week 10

Week 11

Week 16

**End of Trial Follow-up Telephone call**

**Screening**

Screening Visit in Newcastle

**Thank you for taking the time to read this summary.**

**If you are interested in taking part in the AIMM Trial please read the full patient information sheet.**
